# Identification of miRNA precursors in the phloem of *Cucurbita maxima*

**DOI:** 10.7717/peerj.8269

**Published:** 2019-12-11

**Authors:** Eugeny Tolstyko, Alexander Lezzhov, Andrey Solovyev

**Affiliations:** 1 Department of Virology, Moscow State University, Moscow, Russia; 2 Faculty of Bioengineering and Bioinformatics, Moscow State University, Moscow, Russia; 3 Belozersky Institute, Moscow State University, Moscow, Russia; 4 Institute of Molecular Medicine, Sechenov First Moscow State Medical University, Moscow, Russia

**Keywords:** miRNA, Pre-miRNA, Pri-miRNA, miRNA precursor, Phloem, Phloem RNA, RNA transport

## Abstract

Plant development and responses to environmental cues largely depend on mobile signals including microRNAs (miRNAs) required for post-transcriptional silencing of specific genes. Short-range cell-to-cell transport of miRNA in developing tissues and organs is involved in transferring positional information essential for determining cell fate. Among other RNA species, miRNAs are found in the phloem sap. Long-distance transport of miRNA via the phloem takes a part in regulation of physiological responses to changing environmental conditions. As shown for regulation of inorganic phosphorus and sulfate homeostasis, mature miRNAs rather than miRNAs precursors are transported in the phloem as signaling molecules. Here, a bioinformatics analysis of transcriptomic data for *Cucurbita maxima* phloem exudate RNAs was carried out to elucidate whether miRNA precursors could also be present in the phloem. We demonstrated that the phloem transcriptome contained a subset of *C. maxima* pri-miRNAs that differed from a subset of pri-miRNA sequences abundant in a leaf transcriptome. Differential accumulation of pri-miRNA was confirmed by PCR analysis of *C. maxima* phloem exudate and leaf RNA samples. Therefore, the presented data indicate that a number of *C. maxima* pri-miRNAs are selectively recruited to the phloem translocation pathway. This conclusion was validated by inter-species grafting experiments, in which *C. maxima* pri-miR319a was found to be transported across the graft union via the phloem, confirming the presence of pri-miR319a in sieve elements and showing that phloem miRNA precursors could play a role in long-distance signaling in plants.

## Introduction

In eukaryotes, microRNAs (miRNAs) are 20–24 nucleotide-long RNA molecules that down-regulate gene expression by targeting mRNAs, which contain sequences complementary to miRNA, to either translational repression coupled with mRNA decay or endonucleolytic cleavage (Bartel, 2009). In general, miRNAs are produced from imperfect stem-loop structures contained in long primary miRNA transcripts (pri-miRNAs) by a stepwise endonucleolytic processing (Voinnet, 2009; Ha & Kim, 2014). In metazoans, pri-miRNAs are initially processed in the nucleus by a protein complex, the key component of which is Drosha, an RNase III enzyme; the resulting stem-loop miRNA precursors (pre-miRNAs) are exported to the cytoplasm where they are further processed into mature miRNAs by Dicer (Ha & Kim, 2014). In plants, which do not encode Drosha-like proteins, the consecutive steps of pri-miRNA processing into mature miRNA are carried out by the DCL1 (Dicer-like 1) acting in cooperation with a number of other proteins (Voinnet, 2009; Rogers & Chen, 2013). Recent data demonstrate that, unlike metazoans, mature plant miRNAs can be produced in the nucleus and exported into the cytoplasm in complex with AGO1 (Bologna et al., 2018), the silencing effector capable, as a part of RNA-induced silencing complex, of cleavage of target RNA molecules containing sites complementary to miRNAs (Baumberger & Baulcombe, 2005; Rogers & Chen, 2013).

Most plant miRNAs regulate gene expression cell-autonomously, acting in cells where they are produced; however, a number of miRNAs are found to be transported either from cell to cell, or to distant plant organs via the phloem (Marín-González & Suárez-López, 2012). Short-range transport of miRNA in developing tissues and organs is involved in transferring positional information essential for determining cell fate. For example, transport of *Arabidopsis thaliana* miR165 and miR166 from abaxial epidermis of leaf primordia to other cells takes a part in establishing abaxial/adaxial leaf symmetry by targeting mRNAs of HD-ZIPIII transcription factors that specify adaxial cell fate (Yao et al., 2009; Benkovics & Timmermans, 2014; Tatematsu et al., 2015). In developing roots, miR166 transport from the endodermis toward the root axis specifies differentiation of xylem cells in the central stele (Carlsbecker et al., 2010; Miyashima et al., 2011). Besides miR165/miR166, short-range transport is demonstrated for maize miR390 and *Arabidopsis* miR394 (Nogueira et al., 2009; Knauer et al., 2013).

In a number of studies, miRNAs are found in phloem exudate preparations (Kehr & Kragler, 2018; Liu & Chen, 2018). In different studied species, only specific subsets of known miRNAs are found in the phloem, demonstrating a selectivity in miRNA phloem loading suggested to result from expression of phloem-mobile miRNA in companion cells (CCs) (Kehr & Kragler, 2018). Long-distance transport of miRNA via the phloem, at least when biological functions of such transport are identified, is involved in plant responses to environmental cues (Marín-González & Suárez-López, 2012). For example, under conditions of inorganic phosphorus (Pi) starvation, miR399 produced in leaf vasculature is transported via the phloem to roots, where it targets PHO2 mRNA and therefore regulates plant Pi homeostasis (Pant et al., 2008; Lin et al., 2008; Buhtz et al., 2010). Two other miRNAs, miR827 and miR2111, are also shown to be transported from shoots to roots upon Pi starvation (Huen et al., 2017). In a similar manner, phloem transport of miR395 to roots is involved in regulation of sulfate homeostasis (Buhtz et al., 2010). Importantly, as shown in grafting experiments for miR395, miR399, miR827 and miR2111, the mature miRNA rather than a miRNA precursor is transported via the phloem (Buhtz et al., 2010; Huen et al., 2017).

The presence of pre-miRNAs in the phloem has not been analyzed so far, as the reported studies have been aimed at detection of mature miRNAs and therefore limited to analyses of short phloem RNA fractions (Kehr & Kragler, 2018). In this article, to elucidate whether pre-miRNAs could be present in the phloem, we carried out a systematic analysis of transcriptomic data for *Cucurbita maxima* long phloem RNAs. We demonstrate that the phloem transcriptome contains a number of pri-miRNA sequences and that different pri-miRNA subsets are abundant in the phloem and leaf transcriptomes. Differential accumulation of some pri-miRNAs is confirmed by PCR analysis of *C. maxima* phloem and leaf RNA samples. Therefore, the presented data indicate that *C. maxima* pri-miRNAs can be selectively recruited to the phloem translocation pathway.

## Materials and Methods

### Sequence analysis

Primary data of transcriptomic sequencing of *C. maxima* phloem sap (SRX146322) and leaf mesophyll (SRX4058941) were downloaded using fastq-dump of NCBI SRA Toolkit 2.9.0 (http://ncbi.github.io/sra-tools/). Reads quality check was performed by FastQC (https://www.bioinformatics.babraham.ac.uk/projects/fastqc/). Phloem and leaf tissue transcriptomes were de novo assembled by SPAdes 3.12.0 (Bankevich et al., 2012) in “RNA” mode. Transcripts containing potential miRNA precursors were identified by using BLAST (Altschul et al., 1990) with *Cucumis melo* pre-miRNAs annotated at miRBase (Kozomara & Griffiths-Jones, 2014) as queries. Alignments of primary reads with assembled contigs were performed using Bowtie2 (Langmead & Salzberg, 2012) and visualized with Integrative Genomics Viewer (Thorvaldsdottir, Robinson & Mesirov, 2013). All selected contigs were individually analyzed using BLAST to confirm the presence of full-length pre-miRNA region. The local coverage distribution plots were built using “ggplot2” add-on to *R* language, and Shapiro–Wilk test was performed by the language *R* built-in “shapiro.test” function.

### Isolation and analysis of phloem exudate RNA

To obtain the phloem exudate of *C. maxima* (cultivar Big Max), plant leaves were cut out with a razor blade, and exudate droplets appeared on the petiole cuts were collected by a micropipette, immediately mixed with ice-cold buffer (0.08 M Tris-HCl, 4 mM EDTA, 0.08 M 2-mercaptoethanol, pH 8.6), and stored at −70 °С.

Total RNA was isolated from phloem exudate and leaf samples using ExtractRNA reagent (Evrogen, Moscow, Russia) and treated with RNase-free DNase I (ThermoFisher Scientific, Waltham, MA, USA). Equal amounts of RNA quantified by NanoDrop 2000 (ThermoFisher Scientific, Waltham, MA, USA) were used for reverse transcription carried out using RevertAid reverse transcriptase (ThermoFisher Scientific, Waltham, MA, USA) and non-anchored oligo(dT)_20_ as a primer. For detection of pri-miRNAs and TCTP mRNA, specific primers were used (Table S1); 30 and 40 amplification cycles were used for detection of TCTP mRNA and pri-miRNAs, respectively.

### Grafting

Inter-species grafting experiments were carried out using the “side-grafting” method (Tiedemann, 1989). Cut-off epicotyls of *C. melo* with one true leaf were grafted upon *C. maxima* plants with ten leaves. The cut of *C. maxima* stem was made in the region of intercalary meristem of the third true leaf, where the cutoff *C. melo* epicotyl was placed. The incision site was wrapped with Parafilm, and the plants were kept under high humidity conditions until scions started to grow.

## Results and Discussion

**Figure 1:**
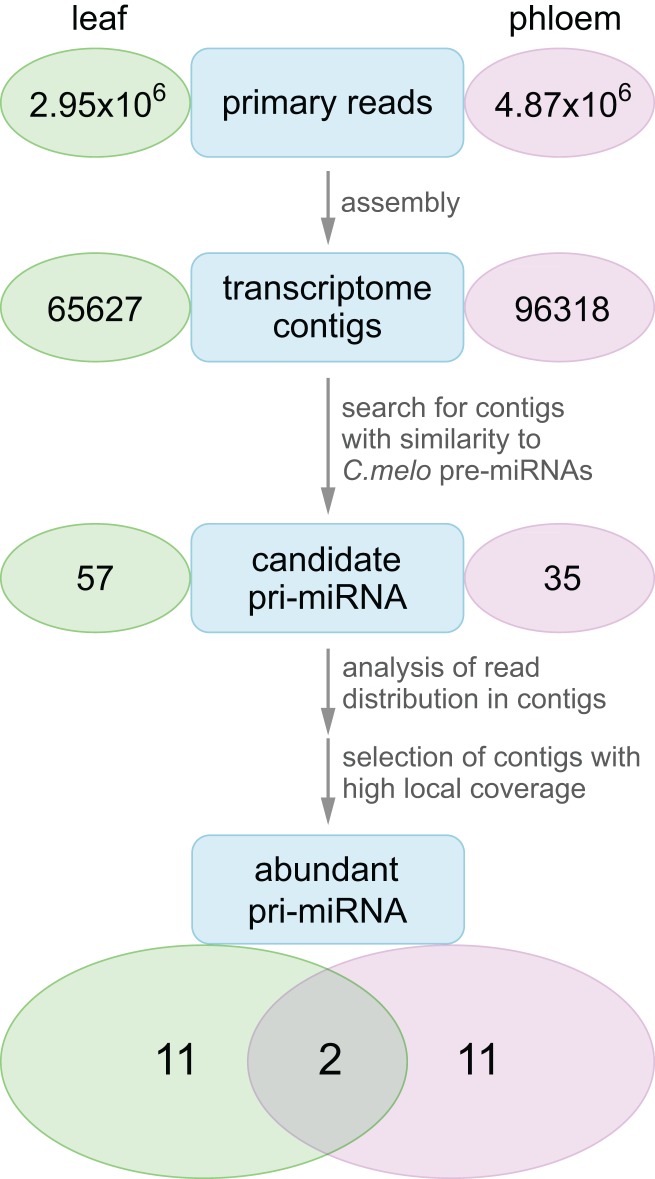
Transcriptome analysis workflow. Numbers in ovals indicate numbers of sequences at each step of the analysis.

To determine whether pre-miRNAs could be present in the phloem, we analyzed a library of primary reads (NCBI Sequence Read Archive Accession SRX146322) obtained earlier by new-generation sequencing of long RNAs from *C. maxima* phloem sap. As a first step, phloem primary reads were assembled into a transcriptome (Fig. 1). As a control, a *C. maxima* leaf transcriptome was assembled on the basis of a library of leaf mesophyll RNA primary reads (Accession SRX4058941). To identify leaf and phloem transcriptome contigs containing sequences of pre-miRNAs, we carried out a BLAST search of the transcriptomes using pre-miRNAs as queries. Since *C. maxima* pre-miRNAs are not annotated, and their sequences are not known, for BLAST searches we used pre-miRNA of *C. melo*, the only Cucurbitaceae species with annotated pre-miRNA. Totally, sequences of 120 *C. melo* pre-miRNA were used as queries. Outputs of BLAST searches were filtered to select *C. maxima* sequences that could be confidently identified as pre-miRNAs. Therefore, only contigs giving local alignments with *C. melo* pre-miRNA sequences with *e*-values less than 1E−15 were selected for further analysis. As a result, 35 and 57 candidate pri-miRNA sequences were identified in transcriptome contigs of phloem and leaf, respectively (Fig. 1).

**Figure 2:**
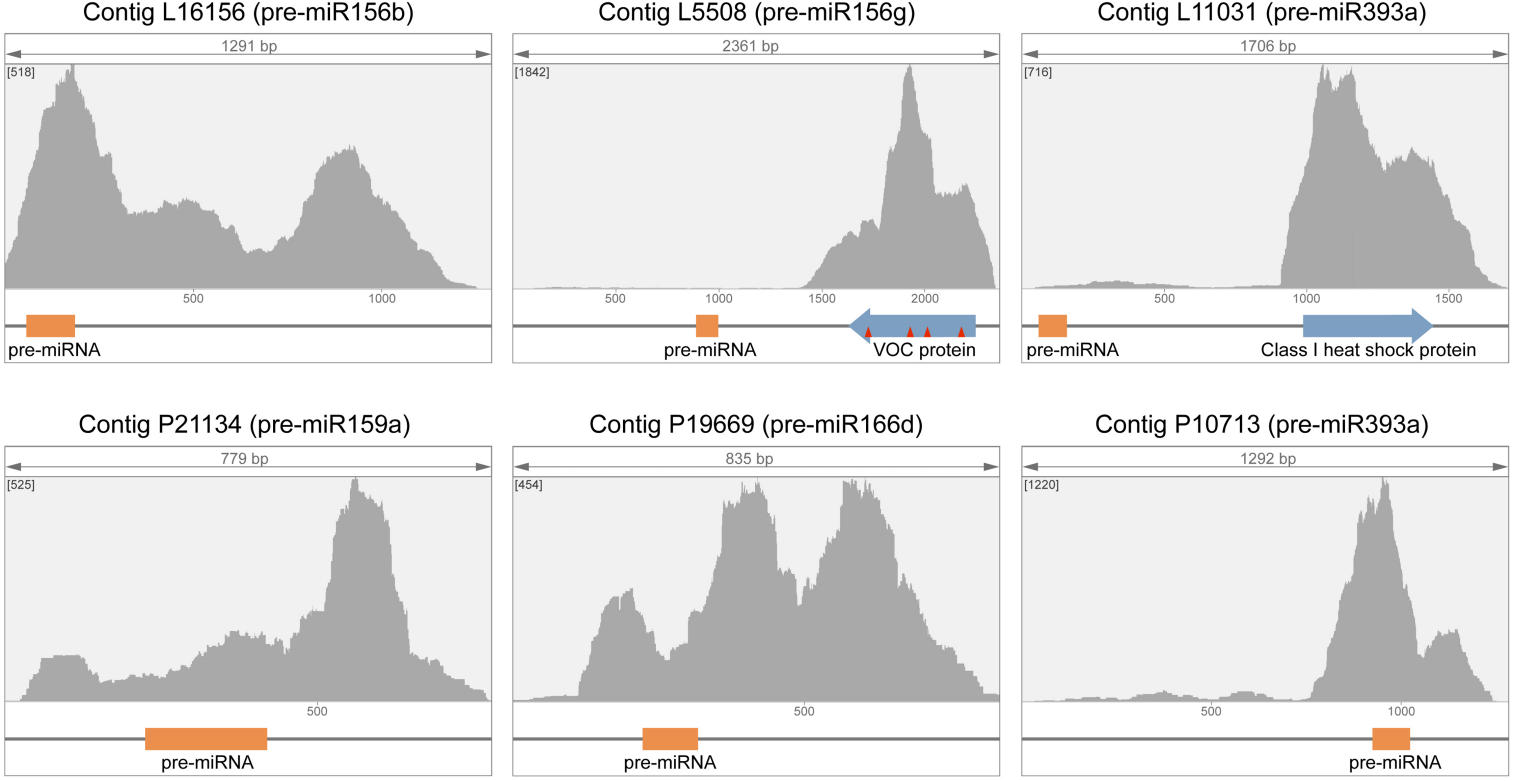
Analysis of primary reads distribution in contigs with highest average coverage (AC) value in leaf and phloem transcriptomes. In presented graphs, the number of reads covering each contig position (*Y* axis) is plotted against the position in contig (*X* axis). Pre-miRNA regions are shown as orange boxes, ORFs are shown by blue arrows. Sites of splicing (exon–exon junctions) revealed by comparisons with respective genomic sequences are indicated by red triangles. The maximum coverage value for each graph is indicated in square brackets.

To identify pre-miRNAs most abundant in the phloem, we further analyzed primary transcriptomic data for candidate pri-miRNAs. The abundance of a given contig in a transcriptome is typically characterized by the “average coverage” (AC) value that is, the average number of primary reads per one nucleotide in the contig. To determine whether a high AC value reflected a high abundance of reads covering pre-miRNA regions, phloem and leaf transcriptome contigs with highest AC values were analyzed in more detail. Primary reads were aligned to transcriptome contigs, and plots of reading depth that depict the number of reads covering each contig position were generated. It was found that pre-miRNA regions were highly covered by reads in three phloem contigs with highest AC values (P21134, P19669 and P10713) and one such leaf contig (L16156) (Fig. 2), whereas in leaf contigs L5508 and L11031 peaks of reading depth were located away from pre-miRNA regions, which had a scarce coverage. Further analysis revealed that the coverage peaks in these two contigs corresponded to protein-coding regions (Fig. 2). The open reading frame (ORF) in contig L11031 was found to encode the annotated *C. maxima* 17.8-kDa class I heat shock protein (Accession XP_022978137), whereas the ORF found in contig L5508 was located in the opposite strand relative to pre-miRNA and encoded an uncharacterized *C. maxima* protein (Accession XP_022987876) carrying a vicinal oxygen chelate (VOC) family domain. Comparison with corresponding *C. maxima* genomic sequence revealed that the contig L5508 corresponded to a single genomic locus and contained four exon junction sites in the polarity of VOC ORF-containing mRNA (Fig. 2). Therefore, likely as a result of inaccuracy in de novo transcriptome data assembly known to be prone to artifacts (Cerveau & Jackson, 2016), the contig L5508 combined sequences of two RNAs of opposite polarities encoded by neighboring *C. maxima* genes and differed drastically in their transcription levels in leaf. Similarly, the contig L11031 likely combined sequences of two transcripts with different expression levels. Therefore, in both L5508 and L11031 contigs, the AC values did not reflect the abundance of respective pre-miRNA sequences in the transcriptome. We concluded that, in general, the AC parameter could not be used for identification of most abundant pri-miRNA.

**Figure 3:**
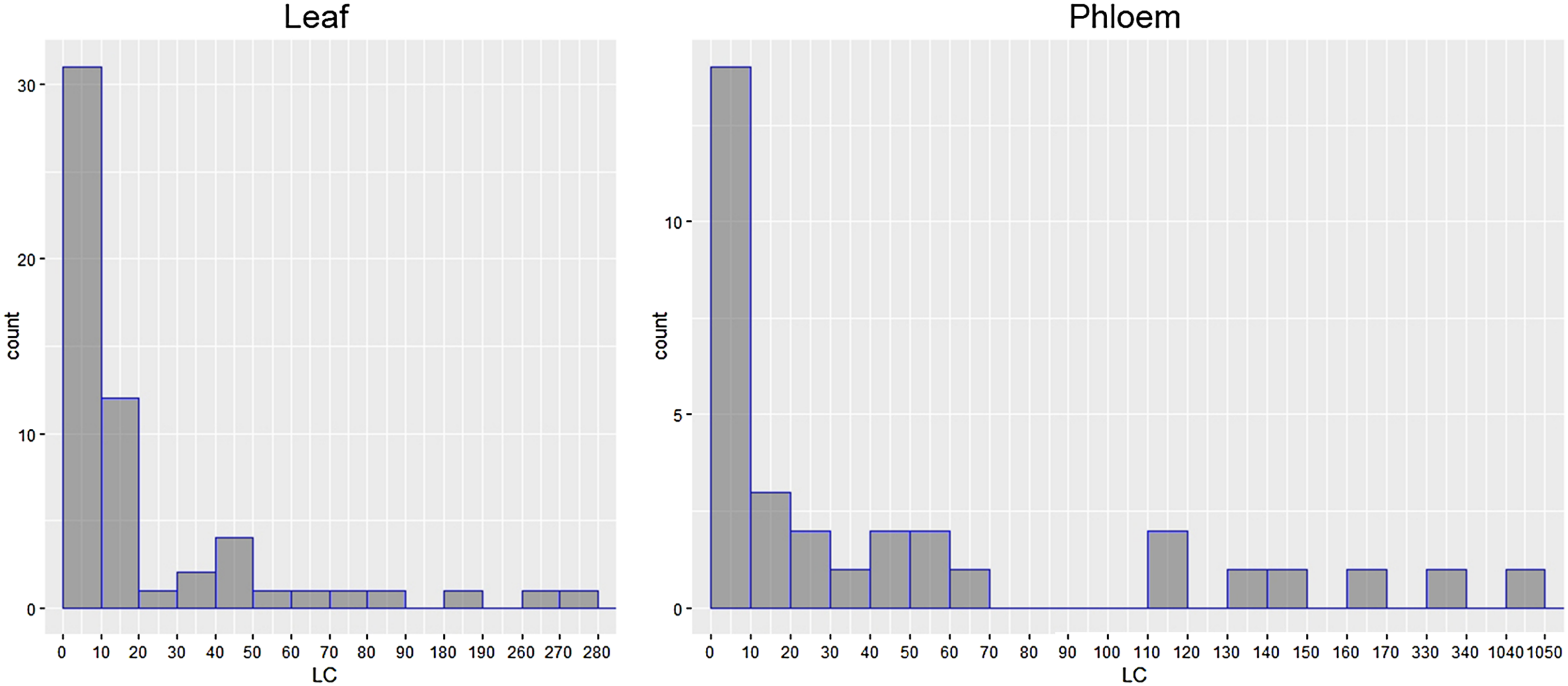
Histograms of local coverage (LC) value distribution showing contig counts plotted for 10-unit bins of the LC value. According to Shapiro–Wilk test, distribution of LC value is not normal (*p*-value = 8.882E−13 for leaf contigs; *p*-value = 3.397E−10 for phloem contigs).

To characterize the abundance of pre-miRNA sequences in transcriptomes in a more adequate way, we introduced the “local coverage” (LC) value defined as the number of reads covering a central nucleotide of mature miRNA in a given pre-miRNA-containing contig. To analyze LC values calculated for candidate pri-miRNA sequences, histograms of contig counts plotted for 10-unit bins of the LC value were built (Fig. 3). For both leaf and phloem transcriptomes, the LC value had right-skewed distributions, and, according to Shapiro–Wilk test suitable for analysis of small datasets, in both cases the LC value distributions were not normal (*W* = 0.41422, *p*-value = 3.397E−10 for phloem contigs; *W* = 0.49014, *p*-value = 8.882E−13 for leaf contigs). Assuming that the LC values likely deviate from a unimodal distribution, we considered groups of contigs with LC > 30 (Fig. 3) as cohorts of pre-miRNA-containing transcripts of high relative abundance in both transcriptomes.

Finally, as a result of all filtering stages, totally 11 contigs with highly represented pre-miRNA sequences were selected for the phloem transcriptome and 11 such contigs—for the leaf transcriptome (Fig. 3; Fig. S1). These two sets had two common contigs (Fig. 1; Table 1), suggesting that the phloem transcriptome contained a specific array of pri-miRNA species.

**Table 1:** Relative abundance of pre-miRNA-containing sequences in *Cucurbita maxima* phloem and leaf transcriptomes.

Pre-miRNA	Similarity to *C. melo* pre-miRNA		Leaf contigs				Phloem contigs			
	Length	*e*-value	Name	Length	AC	LC	Name	Length	AC	LC
miR156b	45	1E−15	L16156	1,300	108.4	268	No match			
miR156c	103	3E−40	L1898	3,420	23.1	73	No match			
miR156e	93	2E−23	L16749	1,261	11.2	48	No match			
miR156i	121	4E−22	L20685	1,010	8.3	31	No match			
miR159a	209	2E−65	L24286	804	14.7	23	P21134	784	72.9	148
miR166d	120	1E−25	L28720	589	1.8	4	P19669	841	98.0	162
miR166e	287	3E−32	L23584	842	7.2	19	P31502	479	20.7	41
miR167b	98	7E−35	L25833	721	23.9	52	No match			
miR167e	107	9E−22	L25150	758	74.8	275	No match			
miR168	218	2E−52	L23344	856	12.5	46	P7902	1,508	10.9	56
miR169f	102	3E−26	L31431	494	28.8	82	No match			
miR171i1	187	8E−43	L9649	1,849	6.0	11	P3867	1,986	48.0	133
miR171i2	192	2E−26	L8895	1,930	24.7	31	P5314	1,784	11.3	23
miR319a	188	2E−70	No match				P20012	828	42.9	53
miR319d	192	1E−53	No match				P20793	797	20.8	36
miR390a	116	5E−24	No match				P37982	364	26.7	111
miR390b	135	3E−21	No match				P38121	362	18.9	49
miR393a	113	9E−28	L11031	1,718	80.8	5	P10713	1,292	102.9	1,042
miR396b1	149	5E−32	L19994	1,055	16.8	50	P29987	513	22.8	61
miR396b2	150	2E−18	L21853	939	14.7	66	No match			

**Note:**

AC, average coverage; LC, local coverage. Gray shading indicate selected contigs carrying sequences highly similar to *C. melo* pre-miRNA (*e*-value < 1E−15) and having high LC value (LC > 30); their counterparts in phloem or leaf transcriptomes (not shaded) are shown for comparison; “no match” indicates that this particular sequence is not found in phloem or leaf transcriptome. Sequences with indices 1 and 2 in miRNA names correspond to different *C. maxima* genomic loci.

In recent decades, it has been discussed whether (or to what extent) the phloem exudate preparations supposed to represent the content of sieve elements (SEs) could be, as a result of destructive methods used for phloem exudate collection, contaminated by macromolecules from other cells and tissues (Atkins, Smith & Rodriguez-Medina, 2011; Schulz, 2017). Identification of different sets of abundant pri-miRNA in the phloem and leaf transcriptomes demonstrated that the presence of pri-miRNAs in the phloem exudate unlikely resulted from a contamination by pri-miRNAs abundant in leaves. This conclusion was further supported by analysis of relative abundance of mRNAs in the phloem and leaf transcriptomes. For example, the number of reads corresponding to mRNA of rubisco small subunit presumed to be incapable of phloem transport was found to be 2,031,410 and 18,233 for leaf and phloem transcriptome, respectively, whereas the abundance of reads corresponding to the translationally controlled tumor protein (TCTP) mRNA (NCBI accession number XM_023119399) known to be phloem-mobile (Toscano-Morales et al., 2014; Thieme et al., 2015) did not show so dramatic difference between the leaf and phloem transcriptomes (56,579 and 201,834 reads, respectively). Therefore, the presence of a particular RNA in the phloem transcriptome did not correlate with its level in leaf.

It should be noted that the abundance of pri-miRNAs in the phloem was considerably lower compared to that of TCTP mRNA. For two most abundant phloem pri-miRNA, pri-miR166d and pri-miR393a (Table 1), read numbers normalized per 100 nucleotides were 213.4 and 214.9, respectively, whereas for TCTP mRNA this value was approximately 100-fold higher (22,729.1). One could assume that the observed lower amounts of pri-miRNA sequences are consistent with regulatory, rather than structural, miRNA functions.

**Figure 4:**
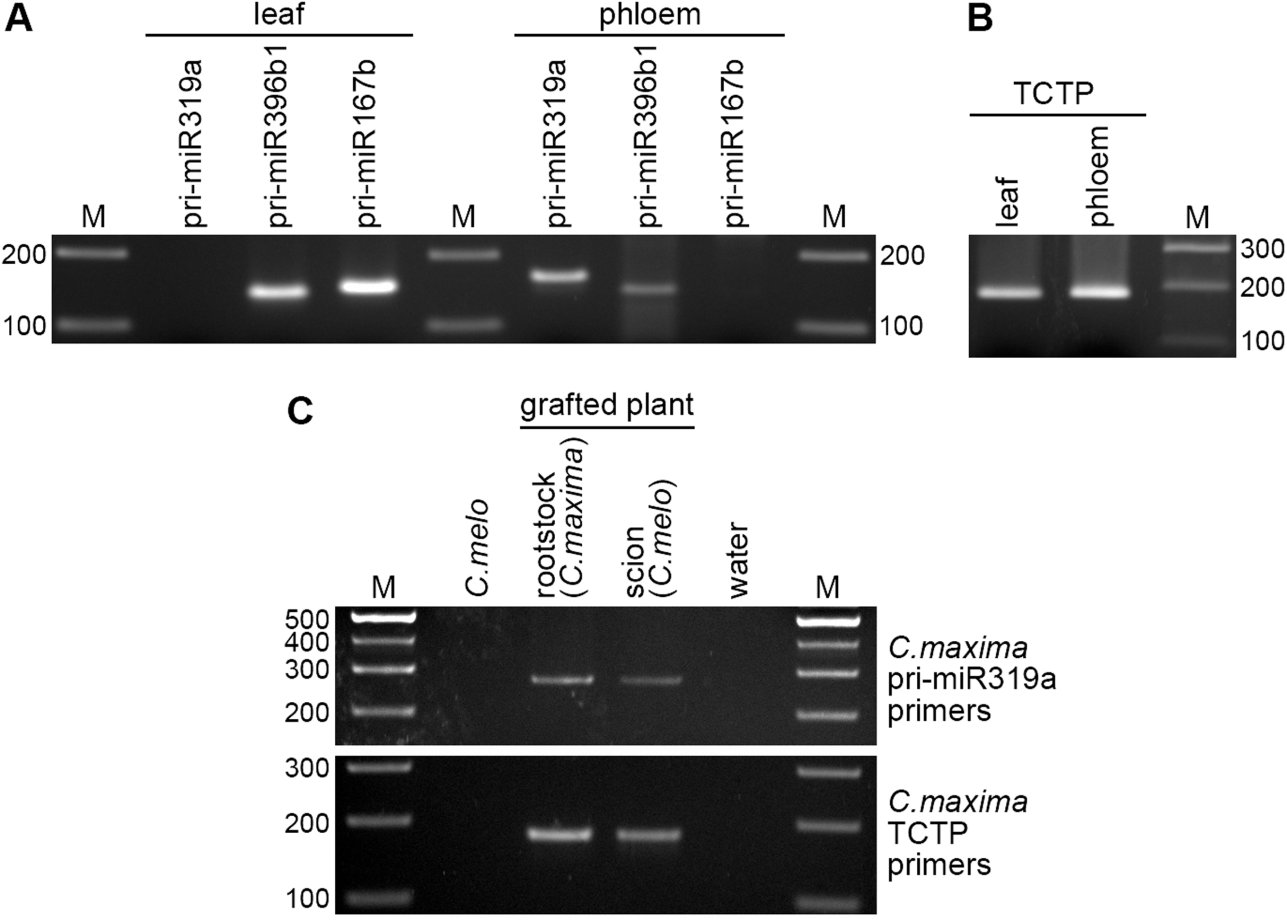
Detection of pri-miRNA in the phloem. Detection of pri-miRNA (A) and TCTP RNA (B) in RNA preparations from phloem exudate and leaf samples. Reverse transcription was carried out with oligo-dT as a primer; pri-miRNA sequences and TCTP RNA were amplified with pairs of specific primers. (C) Detection of pri-miR319a transport across the graft union. Reverse transcription was carried out with oligo-dT as a primer. TCTP and pri-miR319a were amplified with specific primers designed to anneal to respective sequences of *C. maxima*, but not *C. melo*. The sizes of pri-miR319a-specific products in A and C are 162 bp and 276 bp, respectively, as these products were obtained with different pairs of specific primers (Table S1). Water, a control sample without input RNA. M, DNA size marker; lengths of individual DNA fragments in bp are indicated.

To experimentally verify sequence analysis data, we attempted to detect pri-miRNAs in phloem exudate RNA preparations. As a control, RNA was isolated form *C. maxima* leaf samples taken from leaf blade areas excluding main veins. In these experiments, three pri-miRNA of similar abundance were analyzed, namely pri-miR319a that is, present, according to transcriptome analysis, in the phloem but not in leaves, pri-miR167b found in leaves but not in the phloem, and pri-miR396b1 identified in both leaf and phloem transcriptomes (Table 1). Reverse transcription and PCR with specific primers revealed that for pri-miR319a and pri-miR396b1 products of expected size could be detected in the phloem, whereas for pri-miR167b and pri-miR396b1–in leaf RNA (Fig. 4A), validating therefore the transcriptome analysis data. It should be noted that differential detection of pri-miR319a and pri-miR167b confirmed that samples were not cross-contaminated, and that specific accumulation of some pri-miRNA, such as pri-miR319a, in the phloem did not result from their high-level expression in leaf tissues. Thus, based on transcriptomic data and experimental RNA detection, we conclude that a subset of *C. maxima* pri-miRNAs is selectively accumulated in the phloem.

To determine whether pri-miRNAs could be transported over long distances via the phloem, *C. melo* was grafted upon *C. maxima*. Twenty days after grafting, phloem exudate preparations were collected separately from scion and rootstock of grafted plants, as well as from control non-grafted *C. melo* plants. RNA from these samples was analyzed by reverse transcription and PCR with primers specific for *C. maxima* pri-miR319a, which has been confidently identified in *C. maxima* phloem sap (Fig. 4A). As a positive control for RNA transport across the graft union, *C. maxima* phloem-mobile TCTP mRNA was detected. Since the primers, which have been used for pri-miR319a detection in leaf and phloem exudate samples, could not discriminate between *C. maxima* and *C. melo* pri-miR319a sequences, another pair of primers was designed to specifically detect *C. maxima* pri-miR319a, but not that of *C. melo* (Table S1). Similarly, TCTP primers were designed to anneal to the respective sequence of *C. maxima*, but not *C. melo*. Using *C. maxima* TCTP-specific primers, PCR products of expected size were obtained for both rootstock and scion RNA, but not for control non-grafted *C. melo* plants (Fig. 4C). Sequencing of rootstock- and scion-specific PCR products revealed that their sequences were identical to that of *C. maxima* TCTP mRNA. This observation demonstrated that in the analyzed grafting TCTP mRNA was transported from *C. maxima* rootstock to *C. melo* scion, as has been previously reported for TCTP mRNA in heterografting experiments involving other plant species (Toscano-Morales et al., 2014; Thieme et al., 2015). When amplification was carried out with primers specific for *C. maxima* pri-miR319a, PCR products of expected size (276 bp) were found for RNA samples from both rootstock and scion of grafted plant, but not for *C. melo* RNA sample (Fig. 4C). Sequence analysis confirmed that both these products derived from *C. maxima* rather than *C. melo* pri-miR319a. Therefore, these data demonstrate that pri-miR319a is able to be transported across the graft union via the phloem. In should be emphasized that the presence of a rootstock-specific pri-miRNA in the scion phloem exudate could not result from a contamination of scion exudate preparation by RNAs from surrounding tissues and could result only from long-distance transport of pri-miRNA molecules containing in rootstock phloem SEs. Thus, the observed rootstock-to-scion phloem transport of pri-miR319a confirms that detection of this particular pri-miRNA in phloem exudate preparations by bioinformatics and PCR analysis does reflect its presence in the phloem SEs.

Two models for pri-miRNA entering the phloem SEs can be envisaged. First, since the size exclusion limit of specialized pore-plasmodesmata units interconnecting SEs and CCs is considerably higher compared to plasmodesmata between other cell types (Oparka & Cruz, 2000; Lee & Frank, 2018), and a nonspecific loss of proteins from CCs to SEs has been suggested (Paultre et al., 2016), RNAs synthesized in CCs might also escape into SEs, and therefore the phloem loading of particular pri-miRNAs can result from their specific expression in CCs. This model implies that pri-miRNA loading into SEs is a nonspecific process independent of pri-miRNA structure. Second, in accordance with the current view on RNA phloem transport, pri-miRNA can be directed to the phloem translocation pathway by their structural elements serving as specific signals for phloem targeting. Such signals might enable RNA translocation through all types of plasmodesmata, and therefore CC-specific expression can be non-essential for pri-miRNA loading into phloem SEs. Further experiments are required to study these two possibilities. In particular, regarding the pri-miR319a transport, it is important to understand whether the pri-miR319a ability for phloem transport correlating with its presence of in the phloem exudate but not leaf samples results from CC-specific pri-miR319a expression, and whether pri-miR319a structural elements are essential to enable its transport into the phloem SEs. In any case, the pri-miRNA phloem loading is an alternative to pri-miRNA processing and, conceivably, can depend on pri-miRNA ability to avoid processing and exit the nucleus in an unprocessed form. One can hypothesize that such an ability is specified by RNA-binding proteins that can, upon interacting with specific signals in pri-miRNAs, (i) exclude them from a pool of molecules that undergo processing and (ii) direct their nuclear export and further entering the phloem translocation pathway.

The functional significance of pri-miRNA phloem transport is currently unclear. To serve as a signal-transducing molecule, a pri-miRNA should be processed into mature miRNA after delivery to target cells that, in turn, requires import of pri-miRNA into the nucleus, where the processing of pre-miRNAs giving rise to mature miRNA occurs in plant cells (Bologna et al., 2018). Nuclear import of RNA is not widespread; however, a few examples of such a process are documented. For instance, small nuclear RNAs, the key components of spliceosomes, are transported to the cytoplasm to form a complex with a number of proteins that is, imported back into the nucleus (Becker et al., 2019). The tRNA nuclear-cytoplasmic traffic is known to be bidirectional: in addition to conventional flow of tRNAs from the nucleus to the cytoplasm, cytoplasmic tRNAs are imported back to the nucleus (Chatterjee et al., 2018). We hypothesize that pri-miRNA nuclear import can employ one of the pathways used by other cell RNAs. On the other hand, one cannot exclude that, as an alternative, pri-miRNA signaling functions could be unrelated to the production of mature miRNA.

## Conclusion

Mature miRNA are transported through the phloem to contribute to plant responses to environmental cues. Here, using analysis of transcriptomic data and detection of pri-miRNA in phloem exudate samples, we demonstrate that precursors of particular miRNAs are present in the phloem and can be transported via the phloem, potentially taking a part in long-distance miRNA signaling.

## Supplemental Information

10.7717/peerj.8269/supp-1 Supplemental Information 1 Sequences of transcriptome contigs identified as abundant in the phloem and leaf transcriptomes (Table 1). pre-miRNA regions are shaded, mature miRNA sequences are underlined.

10.7717/peerj.8269/supp-2 Supplemental Information 2 Primers used for amplification of *C. maxima* pri-miRNAs and TCTP.

## Additional Information and Declarations

### Data Availability

The following information was supplied regarding data availability: Raw data on assembled contigs identified as abundant in the phloem and leaf transcriptomes are available in the Supplemental Files.
