# Supplementary material for: Identification of miRNA precursors in the phloem of Cucurbita maxima
Source: PeerJ. 2019 Dec 11;7:e8269. doi: 10.7717/peerj.8269 (PMC6911342; doi:10.7717/peerj.8269)
Supplement: Supplemental Information 1 — pre-miRNA regions are shaded, mature miRNA sequences are underlined. [file peerj-07-8269-s001.pdf]

>P3867
AATCCGTGCAATTCGAAGATAAAAGGCCATTCATTCAGAAACTCACTCAGTCATCGGAAAAAAAAAACAGAACAAATTC
TTCTTCCTGTTCTTAGTTAATTAGAATGAGAGTTCCTGTTTCCAATCACCACTCATCCAAACCCTCCTCTGCTCCTACA
CAAAATCCTAAGCTCGACTCCTCCCCCTCCCCCACCGCCTTCCGCCCCCCCACCACCACCGCCACCACCGGTTACGAAC
CCACCTCCGTTCTAGATCTCCGCCGTAGCCCCAGCCCCGTGGCGCCAGATAACCCTCTCTCCTCCGCCGACGACCGTCA
CAACAACAGCCATCCTCTGGACTGGGATGAACGCACTCTCCAGAACCTTGACTGGGAATCCATCATGGGCGATTTGGGC
GTAAACGATGACTCCAATTCTGCTCTCAAGAACACCACCGCCGCCACCACCACCACCACCACCACCACCACCGACGACA
ATACCCACGATCATGTTCCTCACTTTTCCGAATTCCTTCACTCCCAGCCGTTCGATCAAACCGCCCACCTCCTCCCCTC
TGATTTCTTCCTCTCCGAACCATATTCCAATCACTCCGCCCCCATGCTCCACCAGACCTTCGATTTCGCCCCCAATTCC
AACGACCCATCTCTCCATTTCCTCGAAGATCTCGTCGACGCCGCCGATTGCTTCGATTCCAATGATTTCCACCTCGCTC
ACGTCATACTAGAGCGGCTCAATCACCGCCTTCAATCCGCCTCCTCCGCCAAACCTCTTCACCGGGCCGCCTTCTTCTT
CAAAGAAGCTCTTCAATCTCTCCTCTCCACCTCCGCCACCCGCCATAACTCCTCCTCTTCCTGGCCCGACATCGTCCAT
ACTATTAAGGCCCATAAGGCCTTCTCTGCAATTTCTCCGATTCCCCTGTTTTCCCACTTCACCACCAATCAAATCCTTC
TCGAAGCTCTCAACGCTTCCTCTTTCATTCATATAATTGATTTCGACATCGGATTTGGTGGGCAATACGCCTCTTTCAT
GAAGGAGATAGCTGATAAAGCAGAATCAAGAAACGTTGTTCCGCCAGTGATTCGAATTACCGCCGTCGTGTTTGAGGAA
TTTGCGGTGGAGAGTCGGTTGACTAGAGAGAATCTCTGCCAGTTTGCCCAAGATTTGAAGCTCCGATTTCATATTGATC
TTGTGCCGCTTCAGACTTTCAAGACGTTATCTTTCAAATCGGTGAAATTCATGGAAGGGGAGAAGGTGGCGATTCTGTT
AACTCCGACCATCTTCCGCCGTCTCGGGAGTTCTAATGGCATAGCCTCTTTTCTCGCCGACGTGCGATTAGTGTCTCCG
TGCGTCGTCGTGTTTGTCGATTTCGAAGGGTGGTCGGACTCCGGCAAGGCGTCGTTCAAGAGGAATTTGTTGGATAGTC
TGGAGTTTTACGCCATGATGCTCGAGTCGCTGGACGCGGCAGGGGCCGACGGCGAGTGGGTGCGGCGGATTGAGACGTT
TGTTCTTCGGCCGAAGATCTTGGCGGCGGTCGAAGGAGCGGGGAGAATGGCGGTGCCGCCGTGGAGGGAGGTGTTCCAC
TCGGTCGGGATGAGGCCGGTGGCGCTGAGTCAGTTTGCTGATTTTCAAGCGGAATGCTTACTTGGCAAAGTACAGGTCC
GAGGGTTCCAAGTTGGCAAACGGCATGCTGAGCTGGTGCTGTGCTGGCATGAGAGACCTCTGGTCGCCACGTCCGCGTG
GAGGTGTTAGCTGTTAAGAAGTTGAGCTCGTAAAATTGGATAACTACAAATCATGAGTAAAATTACAATTCAAATTCAA
GAACATAACTCAAAATTTTTAAAATTTTGAAATTATATAAATTGGGTGGGGACTAAATTGTAATTTACCTTCTTTTTAT
TTAGAATTTAATTTTCCCAATGTTATATTTACCGTTTTTCTTTTAACTGTTGTAAAAAACAATGTTATCTGAACACTTT
TTGGATAAAAA

>P7902
CGTGTCCTCATCGTCTTCGATTCTGGTTGCTATAAAGGCCCCTGAACCGGCCATGGTTCTTCTCGCTCTGCATAGGGCT
TAATCTCCGTAGTCTCTCTGTTTGCCTCTCATTTCCGTATCCTTAATCTTTCGCGGGCAATATCTCGGCGTTGTTTCTT
CTCAGTTATACACCGGCGGTCTCTAATTCGCTTGGTGCAGGTCGGGAACTGCTTCGCTCCGGTTTGCGTTTGCCGACGG
CCGGCATCTCCTAATCTTCTTTTTCTGTTTTTCTCTGCTTCTATTTTTTTACTTTTTGCTTCATTTCTTTGCCGCCGTG
TGGTTTAGCTTGTTCGGGAAATGTTTGGGCGAATTGGATCCCGCCTTGCATCAACTGAATCGGAAACCGTGGTGAAGAT
TGCTTGGATTCTTACCTTCCTACAGATCTCAGGTACGGAGTTTCTCTTACTTTCGAGAGAGAACGAGTTAATTTAATTG
TTCGCATTTTTTCATGATTGGAGTTGTAGAACTTTGTTGCTAGTTTCGATTTCTCTCCATGACTGAAATTAATAACCGG
TTTGAAAATTACATAATAGATCTGGTCTGTGCTCTGAATCTTATTTAATTATCTTTCTTCCTTTGTCGTCCGCTTTCGG
AGTTAACTACTGAAGAGCTTTAGCTGTGTTTCGCATTGAGGCTGATGTTTGTATATCATAGAGCTAGAGTTATTGTCGT
TGAAGTTATTTGGTTTCTGTTTATTGCTTCCATCCATGAAACACAATGACAGATGTTACATCTAATTTTGGACATAAAA
CTCCGATTCTAATCCGTATGGAATAACGGAACTGTTAGAACTTTGTGTATCATAGATCCAGATTTATTATCGTTGAAGG
TATTTGGTTTCAGTTTATTGCTTCCATCCATGGAACACAATGAGAGATGCTACTTCTAATTTTGACATAAAACTTCGAT
TCGAATCCGTATAGAATAACGGAACTGTTAGAACATTATACTTCATATTCTACTCCTGCTAAAAAATCAAAGGTCAAAG
AAAAAAAAAAGAAAAGGAAAAGAGAGAGATGACAATAATCTTTGAACTGCTGTTTACCTTGTAATAGTGTTTGGTCCAA
TTAATTGATCTGTTTGCTGAGTACATTTTCCGCGAATAATGAGTTTGACAAAAAGAAAGAAAGAAGTGGCGACCTGTTT
GTAAGTTGTAATCTTCTGGTAATTCTTGTATGACTGTAGAATTCCCCGCCAAACCAGGTTTCCTGAATTTTTGTTTCCT
TCGTTTGCATAGTGCAGAGTAGAACTAGAACAATGAAACTTGTATTCCGAAAAAGGGGGGATTTGTTTTCATTTGTCGG
CTCCTGGGCCGTCTGGCTCCATCATTCCATGATAATAGCCTGTGGAAATGGAGACATTCCGATTCCTTTCGTTCGGAAA
TTAAGTATAAGTGAACTGATTGTGTTCCATTGTAATAGGAAATAGCCAGTAGGTTTTGTGGTTGGTAGGTGGTTCACCA
TAAAAGC

>P10713
TAAAGGAAATCTACAAAGAATTTTAGTCAAATTCAACAAGTCTGTAAAACTCATTTTGAAGTCCAATTTTAGTTTAGGT
TTATAAAATTATGGAGAAAAAGTGAATTACAAAACAAACAAACAAACAAACAAAGAGTTACAACACTTCCTTGCATAAA
GTGGTAGTTTAAAGGGGAAAACGTACTTGAGATGCAGAAGCCTTGCCGGAAAGTTTGAGATGGGGAGCGGCGGAGGGCG
GGGGGTTTAATAAATAGAAGGGATTAGTAGCTAAAGGAGGCATCCGAAGGGATAGCATGATCCAAAATTACGTAGAAGA
AATTGAAGGATATAGATAAGTTGGGATCAATGCGATCCCTTTGGATGCTTCCTTTAGTTCCACACAATAGCCTTTCATC
TTCTTTCATCTCCATCCTCTAACTCGGTTCCTAGGGGAACACTTCAATATAAAAAGACCCACTTTAGTGCTTAACCAAC
CTTCCCTCCCCCTTTTATTCATTTTTTTTTATTATTATTTATTCTCTCCCTTTTAATTATAACTAAACTTATTCTAATC
CTTTAATAAGTATAAAAAAATAAATATATCAATACTTAACTTTATTTTATTAATTTTAACTACCTTTAAAGAGTTTTAT
TAGCGTGCACGTTAGTGAAAATTAAGTGAGAGTGTAAAAATTACTACCACTTATTTAAGATATTGACATTAATTTATAT
ATATGGTCAGAAATTATATAATAATCTTGGAAAAGAAAGAAAAGAGAATAGATTTTGAGGTTTCCCCCTTTTTGTGTAC
AAGGTGTTGGTGGAATTTAATGGGTTTGAAAAACATTCCAATTTTGGGAAAATTAAGTTTCGTTATTCCCAATGCACTC
AACCCGTTTGTTATTTTTTCCAGAGAAGGAAGAACAGGAAGAGAAAGAGGAATGGCATGAATAGGCAATGGAATTGTGT
TTATAATCCATGTGATTATGTTTGATTACGTTGAGCCAAAAAAAACCCAAAACCCATTTCATTGTAGTATCTTATGGAA

CACACATAGTTGTAAGTCTCATTATTGTTAACAAATGAATGTTTTGTTTGTGTGATGCAGGGCATTCAGGCTTAGTTGT
GCAGATGGGCATTAATAGTGGAATTTATTTTGAAAACTTTTTGAGTAGAGAAAAGAAAGTGACAAAAATGGTGGTGTGA
TGGGTTTTTGAAAACTTCCTCCATAATGCACAATGGTGGGTACCTTATAAATGGTGCACATGGAAAGACGATGATGATG
ATGATGTATGGTGCTAGATTTTGTCCAC

>P19669
TTAGATGAGGGTAGAAAGTGGAGTCCATGTCACAATGCCCATACCCAACAAAAGCCTTTCCTTATTACGTATAAAAGGA
CCCCATGAAAGTTAAAAATAATGCATGGCCAAATCAAATCTCAATCAAACCCCTTCTCTCTTTCTTTCTTTCTTTCTTT
TCGTTCCATCTTTACACTTCTTTTCCTCTCTCTCTCTCTCTCTTTATGAACAGTTGAGGGGAATGTTGTCTGGTGCGAT
ATCTTTCATCCTTTGAATTTGAAACAATCAAACTCTTTTAAGTTTTGAATGATTTCGGACCAGGCTTCATTCCCCTCAA
CACACCCCATTAAATCTTTCACTTTTTTCTTCAGTTGGGTGGCCTCGGCAAGCCTCTGCTGCCCGTCATACAGGTATGC
TGGTTATATACTTATGCAGGAGGTCTGTGAAGGCTGTCCTAATTCACCCAAGTTTTCATGGCTGTTTTACTGTGTGTTT
TTTGATTGTGTCGGGAAGTTTATTTGAAAGAAATAAGGTGGAATCTACTGCTGTTTCTTTGTTCTTCTGTTGTGCTTAT
ATCCAAGATGAAGTTTTGTTCTTGTTGAGAAACTTTCCCCTCCAGCCCAAACTATAATTGTAGCTACACAATAGAATAA
GAGGGGAGGGGAAGGGATTCCAACTCCAGTCAATTTTGTTCGTTAGAAATGATTTGGAAGATGTAAACCCGAAGGGAGT
AGCTGTCAAGAAGCGTACTGTAGTGTGCTTGATTTTGTTTCTAACGGGTGCCACTTTGGTTCATTCATCTTACAATTGA
TATAGCTGATATGACGGAAGAATATCAAATCATTACTAAACTAGAGAAACC

>P20012
CCCTTTTCCTCATCTTCCTGGGATTCATGATCAGTTTCTTCATCTTCGTATCATTATATTCTTTAAAGGGTCTCTTTTT
CTCTCTGATTTTAGGGTTTTTTCGGTTGTTGGCGTGATCGGATTTGATGTTTTGTGAGGGTTAAGAGAGCTTTCTTCAG
TCCACTCATGGGTGGCGGTAGGGTTTAATTAGCTGCCGACTCATTCGTTCAAATACTGAGTCAAAAACCCGACTCTCCG
ATTCAGTAAACGAATGAATGATGCGGGAGACAAATTGAATCTTAAGCTTCCTGTACTTGGACTGAAGGGAGCTCCCTTT
TCTTTTACCCTTTTGATTTTATCCGTTTTTTTTTTTTTATCGAAATCTTCGTTTTATCCGTTAAACGATCGAAGACCCAA
TTTCATAATTACGGTATTATGTTGAAAAGATGTTAAAAATCTCGTCTTTTTTATGGTTTTCACCAGCATTGATGGTCTG
CTTGGAGTCAGATTTGAGTAGGAATTACCCATTTGAAGAAAGTTTTGAGGTAAGATAAGTTAAGGTTGATTCATTATTG
GGTTAATGGGTGGATTAGGGTTCTTGAAAATTGAAGGAATATTTTTTACAGAGCAGTAAAAAAAAAGGGTTTTTGCTGG
ACAGACACGAGCTTCGATGCCATAGCTTCTTGGGTATTGGCCATATTGCTGCAACAAGTTCCCAGACATTTTATGTTAC
CACCACCCTCTTCCCATTCTTGGGTATCGGTCTTAATGTTTTGTTTTTGTTTTTGTTTTTGTTCTTCTCTTTGTCTGTG
GTTAGTTTCTTTTTTGTGTTGTTTAAGGTGGATTTAAG

>P20793
TCTCTCTCTCTCTCTTCCTCAGATGACTTGGGTTAATTTAAGCCGTTTCTTGTGGTTTTAATTGATGGATTTGTTGGTG
GGTTTCAATATTTTGAATTAGATTGTACAAAGGAATGAAGTTAATTTATTAGCTTATATGGAGTAAATTAAGATGTGGG
TTTCATAAGTATTGTGTATTGTGTTTATGTGCAGAAAAAGAAGGAGCTCTCTTCAGTTCATCCCGAGACAGTAGAGGCT
CAAAAGGTTGCTGCTCATTCGTTAGTTCAATAGCTCATTATACTCAAAATTTCCACCGTTGAGATGCCGAAGCTGTGAG
ATGAGCTCCTGATCTAACGATGGAGGAGCGGTCTTTGATCATCACTGTCATGTATTGGACTGAAGGGAGCTCCTCCTTA
ATTTACTTTTCACACGCATACTACAACTAACTCATCCTTTGTCGAGAATGATTCAGTAAGTAATAGTTAAAAAGCTTGT
TTTTTTTAACTTTTTACAATGACCCTTAATTTGTGTAGGAATATCTGTCTGTTAAATTTGTGTGGTTTGATAGAGATTT
GAAGAGAGCAGAAGCAGTTAAGGAGGGTAGCTTTCTCAGGGTCCAAAGCTTTGGGCGATGTGTTGCAACTTTGTGAATC
TTCCTTTTAACACTAATTCTCTTTGTGGTCTTACTATCATTATTTTTTTTTTTCTTTTTAATTCAAATATTGTTGAAATT
AAATAATAATTTAAATTTTATTTGTTTAGCTATAAACATTAATATACTTTATGCATTAAAATTTTAAATTTAGATGTGA
AAATGAA

>P21134
CCCTTTCCTTTCATCTTCAACCCTTTTCCTCTTATGAATTTTTCCTTTCATCTTCAACCCATATCTAATGGCTCGTCAT
CTACCTCTAGGGTTTCTAACCCACATGGGCTTTGTTCTTCCTGATCTAAACTGGGTTTTTGCTCTTCTTTTCTGGGTTC
TTCTTGGATTTTTGATTGATTGAGTGTTTGGTTTAAAAATTTAATCTTTTTGGGATTAGGGTTACAGGCAGAGCTCCTT
GAAGTCCAATAGAGGGTTGTGCTAGGTTGATCGAGCTGCTGAGCTATGAATCCCTCAGCCCCTATCCCTATCTCATCAA
TCATATCTAAAGCTGATAGGCCTGTGGCTTGCATATCTCAGGAGCTTCATCAACTGCTTTTGTTAAATCCTTGTTTGGA
TTGAAGGGAGCTCTACATATTCTCTCTAATCATCTTGTTATTACCTTTTCTTATGCCCTAATCTTCATTGACTTGAGTT
TCTTGTTCCTATTATTTGTTATGATTTGATGAAGGTCTGTGATTTTTGCAGGGGATGGAATGGTTTCATTGTTGGATCT
TGCTTAGATTGATGGGATCTTTTAGAACATGTTGTGAAGAGATGAATGTCTTTTTGTAGTGTTGGAGTGTACGATGTTG
CTTTGTAATATAGCCATTTTCAGGTTCTTGGAGATTTGCTAATGGAACCCTTTGTTGTTATTAAGCAATCATTCAGATC
CTTTAGCTTTTGGCTATTGAGTATTGATGAATAGTAATGGCAACGATGAACTATAATCTATCCTATTTTCTCG

>P29987
CTCACTCCTCTCCTCCAGTCTCCGCCTTCTCTCTAGGGTTCGATACCCTAATTTTTCTTTTCTTCGTTCTTCAGAAATG
GCTCTGTTTGTATTCTTCCACAGCTTTCTTGAACTGCATCTTCCTGAGCCAAATTCGCTCCTCTCTCTCTCTCTCTCTC
TCAAAGCGTTCTTTGTTCATTAACTTTTGCGGTTCAATAAAGCTGTGGGAAGATACAGACAAGGTCAACCAAATCGGTA
ATCCACAGCGAAATTCGCCGCCTACAATGACCTTCAATCGCCGGCGCCGTCGGTATATTTCTGTAACTTACAAAATCGT
ATGATGTAATTTTCCCCTTTCTTAAGCTGTTTTTGTGCGTGTTTGTTTGTTTGTTTCTTAATTTGCCTTTTCAGAAACG
GTAACTTTTAGGTCAATTTCTCCCTGAATTTACTCGGAAATGTGAGGTTTCAATTCGATTTCGATTTAATACCTAATCT
TCTCCGTTGATAGCAGAAACTATCTTGAATGGATTCAAG

>P31502
CTTATTCTCTCTCTCTCTCTCTCTCTCTCTAAGCCTCTGATTTTGAGCAGATGGGAGAAGAGAACATGAAGAGTTCTCT
TTTGATTATTATTGCAATGAGATAGGCAAAGGGGTGTTTGGAATGGAGTTTGTTCCGAAATCTTTGTGTTTCTTCGTTT
AAAGGGTCGTTCTTTTTCGTTTATTTTGTGTCATTTTTTTATCCTTTGCAAGGTTTCTTTCTTATGAGGTTGAAGATTG
TGTTATTCTATGTTTTTTCGAACTAAAAGTTGTGTTCTTCTTCTTCTTCCTTCTCGTCTTTGAAAAGTTCGTCGTTTTG
TGTTGATTGAATGGAAAAAATGATCTCGGACCAGGCTTCATTCCTCACACGTAGCTTTTCCTTGGAAAATTTTGGATAT
GGGAAAGATGAAAGACCTTTGATCAACTTCTGGTAAGCTCTCTTGAATCTCTCACGAGGATCATATATATATATATATA
TATAG

>P37982
CGCCTCGCCCGCCGACATGGTGCTCCGGCAACCGGCAGTAGCACGGGAGAATCCGTCAAGCTCAGGAGGGATAGCGCCG
TCGAATGAAACACCACCGGAATGTTCATTTCCGCCGCCGCTTGCCGGCGCTATCTATCCTGAGTTTCATGGCTTCTTCT
TGCTTTTTCCAGCTCGGTTTTTTACAGGCATTAGAGGACACGTGGACGGTCGGATGGAGAGGAGTGGAATGAAGAAGGT
TAAATAGCAATGGGTACATGCAAGACAACATTGCGTTTTCTTTATAAAATTAATTCCAGAAATTTTAGATAATGTTGAA
ATATGGACTAAATGAAAATTAATTATAAATAATAGATTATATCCGTCG

>P38121
AAGCCAGGAAAGCAAAAGAAATGAGAGAGAGAGGGGAGGGTTTAGTTTGGGAGAATCTGTAAAGCTCAGGAGGGATAGC
GCCATGGTTCGTATCAATAATAATATTATTACACTTCCATTTTCTTTCAATGGCGCTATCCATCCTGAGTTTCATAGCT
TCTTCTTACTCACTCGTCTTCTCCTTCCTCTTCCTCTTCCTTTCATAGCCTCTCGTTAACGTTTTAATAAAAATCGTTT
TTTAAGCATTTGAAAAGTTATTCTAAATGGAATGATTATGTCATTACATTCTTCTTCTTACGTTCATAATCGTTAAATT
TTCTATTGATTGTTTGTAACGATTGGGATGTTGATTATATCACCCG

>L1898
AAAAACTCCACGAAGTCTCTAAGCCTTTAGGCATGATTATATTGCTCACCTACCAATAACATTAAAAATGCCTCGCTGT
TTTTGGAATTTCTGCTTCATAAAATGACACTCTACAGACAGAGACAGAATTACATTCACCAAAAAGATTCATAAATTTG
AACCAACTCACTCATTAGTTAAGATATCTACTACTTTTTTTTGAAGTCGAAAATTCAAATCTACACCCAGCATTTGTTG
CATTCAAAAAGTCACGAGGGTGGAAACAACAATAGAGCACTATCAGTCACTTCGGGTTTATGACCATAATCGAATGTGT
TAATTGTAGCATGGTTCCAAAAAGAATCTCCCAAGTATAACCTTAAATGATTTTTTTTAAGGTATGGGTTTAATCACTA
CCAAACTACTTCAAATGGAAATGGAATGATGGAAGGAGGAAGGAGTAGCAAAACTTACTTCTTTCACTTAATATGCACT
CCAAACAATTTGAAACCCTCAACTTCAGCATCGCCTTTTAGTCCGTCTTTGTTCTTAGGCGATAACAATTCTTTGGCTT
CATTCTCGACCGAGCTGGCACCATTACGATTTTTAACATCTACCACACAGAATGTAGTTTTAGCATCATTTGTCTTTGC
TGCTTCACACAAGTAGAAAGCTATTGTGTCATTAGCTTTCAGTTGTTTTTCCTTCACAAATCGATTCCAACCACGTGTG
AACACATAGCTCTGGCTGCTTTTCCAGTAACAGTATCGAAATTTCCACTGTCTCATTGTTTTGTCAAAAAATATCAGCT
GCACATCACGATCGTCATCGGCACGTTCTACGTTTTCTAACGTGCTAGCTGATACGTGAGGAAAGTATTTAACAGCATA
TTTCTTTGGGATGACCAATCTGTTTAACTTACCAACATCGCTCGGTGTTAGCTCCTTTTGAAAAAGTTGCTTAATAGTC
CCTTCATTGTTATTTATATTTGCTGCAGAGAGGCCGGCTGATGTTTGTGCACTTTCAGAACGGTCCCTCAAATACTCAG
CAAATTTGGTTGTGTAAGAACCGTCCTTTAACATATTAAGCAAAGCTTCTGTAGTGTAAAGTGTCTGAAAGTTTGGCTC
TTCAATTGTGACCTTGGTCCAAGGAAAGTTTCTATGACAATCTCCGCTTCTGATTCTGATAGCTGCACTATCATAAGCC
ATGGCTGCCTCGTTTTCTGACTTGAATGTACCAAGCCAAATACGCTGATGATTGGCGTAAATTTGAGCACCCCAGTGGC
CGTTCTGTTGAGGCACAACACCCTTGAATTTTGAGGTCAAACTCCTTCCTTGCTTACTGCACCCATTTGCAGGGAATGG
GCAAGTGATGCAGCTTGAATCTGAACCTTCTGCAATTGCATTCACCCTGGTTTCTGAAATAGTACTTGGCCACTTCTGT
TTCATGATGGTTCTCGCAACCGGACAGTGGGGGTGGTGGTTATGATAGCGAATCTAAAGTGAAGAGAAAAATAAGCAGG
CTGCTGCCTCCCAACGTCAAGCAAGTGCTGAATGGGGAAATTTTCCGGTTTGGAAGCTCCATATCTGGGACTCGAAAAC
GGCAGGACCTTCAGGCATCCAAAACGAGAGATTGTTGCCATATTCAGCACTCGAGTCGGTCCAGTCTCACACCCTTCTC
CAAGTAGTACCCCACGACGGCAAGAAATTTTTCGTTTTAGGAAAGTAAAAACCAAAACAAAATCTGCAAAACTCTTCAA
CGACGAAAATAGTGCGTATATAAATATATCCCAGATTCCAATATTCTCAGCAATGGAAGAAATATTTGGTCAGATAAGG
AATAACCCAAATCTCAGGAACCAAATAAATAAAAATAAAAGCACAGTGTGTGCTTAATATCAAAGGAAACCCCAAAAGA
TGGCGGGGAGTTTGAGATTTAGATAGGAACAGTAATCAGGGTTTTTCCACTAAACAACAACGATATTCGAAGGATTTTT
CTGTTAAATATGAATAGTAATTGATTTGTACCATCAAAATTACATAAATATATATATATATATATATATATATAAGCAGGA
AAGGAGCATCAAAAGCCGTGCTATCAAGTTTCCATGGAAATCAATGTCAAGAAAAAAGAAACAACACCAGAAGAGTTTC
CATGAAAAAATCAGAACCACAGAGAATAAAATAATAATATTGCAATAGATAAGAATAAACTGACAAATTAAGAGTGTGA
TGGCGAAAATTGTACAATACAGTGGCACCGCTTGGATGTTTAGAGAGGCCGCTTCAACTCCGCCAGTGTTGTTGTTCCA
TTTGTCCATCAGAGGATGAAAAATGAAGGGGGTACCCAATATAGAGAGGAACGGAGCAAGAGGACATCTGGCCCCTGAC
AATATGAACCAGAGAGAAGAAAGAAAGAAAGGGACATTGGTATAGAATAGGGTCTTTGATGAGCGAGCAGCGGGCAGTG
AGAGTAGAAGAGAGAGAAGAAGAGATCAGAAGGGAAAATAATGGGGAAGCTGACAGAAAGAGAAGTGAGCACGCACCCG
CAGAGGTATGCTCTTCATACATTTGGCTGCAGCGTGTGCTCACTCTCTTCTGTCACTCTCTTACCCTTCTGGTCTCTCC
TTCCATATTCCATCATAAAATCTCTCAACAAAACCCAACAATTTCCCTTCACTCATCTAATCCATCACCTTAAACCCCA
CCCATTTATGGAATCAATAAACCCATAAAAGAGATAACCACTTTAGCCCACCCCATGAGAAAAAATTTCCCTTCTTTCT
TTGTCAGACATTTCATCATACATCTTCTCTGCATCCTTCCTTCCATATTCTTGTAATATTAAACTCCTCTCAACTCTCA
TGTATTTCCATCATCGACACCAGCAGAAACCGGCAAGGAGAAAGCGAAATAGGGTTTCGCTATTACATCCATAGATATA
CCCATTTCACACGCCATCAAAAGCAAAAAACAACAAAACAACCCTGACATCTTAACAACTTTTCTCTTTCTAAAATACG
CATCTGGGAAAGCCACCAATGACAATTCCACCACCACCACCACCGTTGGTGGAGAGCGAAACAGATCAGAGGGGCGGTA

GGGACAGTGTTACAAAATATGTACAGAGCGCCAAATTTACGAGCGGAGGGGAAGCAAAGGACGAAAGGAAGAGGACATG
ACAGTCCATCGAGTGGTTTTTTGTGGGACCGTTATCTTCCTCAACTTGTAGCTGTAAGGTAAGCATTGTAAATGTGGTC
TACGTGGACCAATCAGATCCCGCCACTGACTTCATGCCCTAATCCTCAATGCTTTCCTTCCGCCATTTTTAATTCAACC
ACATTTTCCACCGTCCGATTTTCTTCTCAATATAATCTCTTAATAAATATATTATATAATTTTTTTTTTTTTTAAGTAT
ACTATTATTGTAAACTAATCCCA

>L8895
AAGAAAACCGACTATATATTAATATTAATATTAATTGGAGAACGGCGGGATACATACCATTTTATGTACGTCCCTACAG
TTAAACCCAAACCCCTAATCCCAATTATCGTAAATTATAATTTTTAATCAATTTTGATACCCTCAATATCTCGGGTAAT
TAAGCTCTCAACACCTCCACGCCGACGTGGCAACCAGAGGCCTCTCGTGCCAGCACAGCACCAGCTCAGCATGCCGTTT
TCCTACTTGGAACCCTTGAACCTGTACTTTTCCAAGTAAGCACTCCGCCTGAAAATCTGCAAACTGGCTCAGCGCCACT
GGCCTCATCCCGGCGCCGTGGAATACCTCCCTCCACGGCGGCGCTGCCATTCTCCCTGCTCCTTCCACCGCAGCCAATA
TCTTCGGCCGGAACACAAACGTCTCTATCCTCCGCACCCAGTCCCCGCCAGCCACCGCTGCGTCCAGCGACTCTAGCAT
CATGGCGTAGAACTCCAGGCTATCTACCAAATTTCTCTTGAACGACGCCGCGCCGGAGTCCGACCACCCTTCACCGTTA
ACGAACACGACGACGCAGGGCGACACCCGTCGCACGTCGGAGAGAAAAGACCCTATACTGTTTATACTCCCAAGACGGC
GGAAGATCGCGGGCGTTAATAAAATCGCGGCCTTCTCCCCTTCCATGAACTTGACCGATTTGAACGACAACGTCTCGAA
GGTCCGAAGCGGCACCAGATCTATATGAAACCGAATCTTCAGATCCTGAGCGAACTGACACAGATTCTCCCTAATCAAC
CTACTCTCAATCGCAAATTCCTCCGGCACCACCGCCGTTATCCGTAGCACCGGCGGAATCATCTTCCTGGACTCTGCCT
TCTCTGCAATCTCCTTCATGAAAGACGCGTACTGCCCTCCAAACCCAATATCAAAATCAACAATATGAATGAAAGACGA
ACCGTTAAGAGCTTCAAGAAGCGCCTGATTGGTAGTGAAGTGAGAGAACATGGGAATTGGAGAAATTGCAGAGAAGACC
TTGTAAGCCCTGATTGTATGAACAATTTCGGGCCAGGAGGAGAGGCGATTGTGGCGATTGGAGGCGGAGAGGAGAGATT
GAAGAGCCTCTTTGAAGAAGAAGGCGGCCCGCTGAACGGGCTTCCCGGTGGAGGATTGAAGGCGTTGATTGAGGCGCTC
CAATATGACGTGGGCGAGTTGGAAGTCATTGGAATCGAAACAATCAGCGGTGCGGACTATGTCTTCAAGGAATTCGCAG
GAGAAGTTGTTGGAATTGGGAGCCGCCAAATCGAAGGAATTGAAGGTGGGGGTGTGGTTGGAATAGGGGGGTTCAGAGA
GGAAGAAATCAGGGGAAACCAGATGGGTTTGATGATCGAAGGGGTGGGAGTGGAGGAAATCAGGGAATTGAGGAACATG
ATTATTGGGATTGGGATTGGGATTGGGATCGGGGTTTGGGTTTTTGGGAGCAGAGTTGGAGTCATCGTGCAAGCCCAAA
TCTCCCATGATGGAGTCCCAGTCGAGGTTGTGGAGGGCCTGTTCATCCCAGTCCAGAGAAGGGTTGTTGTTGTTGTTGA
CGTTGGGACGATCGTCAGTGGGGAGGAGTGGAGCGGAGGAGAGGGTGTGGGGGTCAGGCGCCACAGGGCTGGGGCTGCG
GCGGAGGTCGAGGACGGAGGTGGGTTCGTAACAGGGGTTGGTAATATTGGGGGTGGAGGTGGGGGTGGGAGTGGGGGTG
GCGACGGCGGAGGGCGAGGAATTGAGTTTAGGGTTTGGAGAAGAAGAGGGTATTTGGTGATTGGAAACAGGAACTCTCA
TAGCTAGCTAAGATGAGAAGAAGAAGAAGAAGAAGAAGAAGAACAACAACAACAACAACAAGGGCTGGGACTATGTCTT
CAAGGAATTCGCAGGAGAAGTTGTTGGAATTGGG

>L16156
TTCATCACATTCTCAAGGACTTAAGAAAAATCAACTCAATTCTTGGCCTTTTTTTTTATGTATCGAAACAAATACTGATA
TGAATCATAACATCGGATCCTTATCTATGTTCATTAAGAGAGAGAAATAGAAGGCAGTTTGAATATTATGATTCCAAAG
TTGGGTACATGTTATGAATGAAGGAGATGAGTTAAGTGAGTGAAAGTGGGTGGAGGGGGGAGACCACTAGGGCAGAGTC
GAGTGGAATCTTTCCTCTTTTGTGTGCTTCTTATTTGCTCCAACAACACACACAAAGGGCTGTCACTGTCTCTCATTAG
CTACTCATCAATCCACCAACAAAAACAACTCCTTCTTCATGCATAACAAAGAACACTAATAAGGATTATGTCTCTTAAT
CCACATGCATAACATGGTGAAACATTAATACATCATTAATATCAATCTTGTCTCAAACCCACTGGATCAAGATGTCTGA
GAAGGATCCGTGTGAGCCTCGTTTTTTTGCTTCTCTTTTGAGGAATATTCCTCTTTACTCTCTTAAGATATGCTGCACC
CCTTCATCATTTCCTGCATTCATTTCCAAACACAAGAACAAACAACATCATCAACTCATTTGAAAGGAAATTTTTAATC
TTATACAAATACATCAAGTGTATCTATGATCCTTTCTTTGCTCAATTCACCAAAAAATGAGTCGTTTGACCGTTAGCAG
CAGATATTGTCTCCCCTCAAGGTACTATGAAGAGGGTCTTGCTTCATCCGGTGACTGACTCTGATAAGGAATGTTTCGT
TCCCTTGTCTAGCCGATATGAAACCTCACAATCCATAGGAGGACCAGAAGCGTTAGAAGATGAAAAGGGAAAGTTGAAG
AAAAGATGTAAGAATGTGAAACTCAAAACCCTTGCCAAACAAGACTTGAAAATTGCACAATTTGAACTAAAACACAAAA
CACCCAACACAGGAATCAGCTCATTTCTTAGGACAAACAGCTCACTAATCGCTCAAGTGACACAAATTCACAACTTTAA
GATAATGCTTGAGAGTTTTAAAAGACTTTTACATAGAACAACACATAAAATTAACTACAAATCAAGAAAAAAGAGACAG
CAAAGCTAAAGAAGGTGATGACAGAAGCATAGAGAGCACAAATGGAGTGAGATGCACAGTGGTCTGTAAAATGTCTATC
AAAAACAAAAGAAATTGCATATCATCATCTGTGCTCTCTATCTTCTGTCAACAACATATCACCTATATGTTCCTCCTTC
ACCCCCAAACGAAGAACAAGTAATAAACAAAGAAAT

>L16749
CTCTATGCTTCTGTCAACACCATATCACCTAAATGCTTCTTCTTCTTCTTCTTCTTCTCTTTCTCTCTTTTTAGTT
TGGTTTATTTGTAAGTGAAGTTTGGGGTTGAATTAAAGGAGGAAGCATTTAGGTGATATGGTGTTGACAGAAGATAGAG
AGCACAGATGATGATATGCAAATTTCTGTATCTCACTCCATTTGTGCTCTCTATGCTTCTGTCATCACCCTCTTCACCC
TCCTTTAACATCCCATCTTCACTTTAGTTTTGTGTTTGATTCTATGTAAAAGGTTTTAAAACTCTCAAGCATTGTCTCT
ATGATTCTTTGTATATATCCATGAGCAATTAGTTGTGTTTGTAGCCCTAAGAAGTGTGCTGATTTGTGTTGCTGGTGTG
TTTTGTGTTGCAGTCCAAATTGGGCAAGGGGATTAAGAGATTTGAGGCACATAATGTAATGACCTGAAGCCACCACTAG
TAGATATTGTGAGATCTTATATATAAGAACCAACCGACCCGTTAGATAAATTTTCAAAAAAAGTAACCTTTTGGATACG
TTCGGCAAGTTAAGAAAGTTCCATGAATACACTTGGTATGTATATGTAAAAACTCATTTTTTCCCCTTCCAAATGAGTT
GAAATTGTTGTTTTCCCTCTTTTCTTGGCAATTGATGCAGGAAATGATGAACAAGAAGAAGATGATATGCCAAATGATA
TTAAGAGAGTAAAGTGAGTATTCCTCGAAGGGGGAAATGAGGCATGCACTGCCCACTTGCTTGATTGATTTGTGGTGCC
AAAATTGATTCATATAATGTTGTTATTCATATTATCCAACTGATTTTGTAATAATAAGGCCTTTCATGTTAACATGGAG

GGAGCTTTCTTTAAAACTATTAGGGTTTTGTTCATTACACAAAATAGTGAAAGATTTGGAACTTTTAACCACAAAGTTT
CATGAGGATTCACAAGCTTTTGTTGGGTTCATAAGGAGTTAGTGAGACACTGACAGCCATTGTCTTTTTGAATCAAATG
AAAAGAAACACACAAAAGAGGAAAGATTCCACGTGCTCTTTACCCTTTGTGGTCTCCCCCCTTCCATCCACTCTCATCC
ACCTAGTGATAGCCCAAGCTCACACCCTACTGGTAGCACCCAAGCTCACTTTTAGTAGACACTGCTCTGACAGAGCCCG
TAGAGCACCCAAGCTCACTTTTAGTAGACACTGCTCTGACAGAGCCCGTAGAGCACCCAAGCTCACTTTTAGTAGA

>L19994
AAAAGGGGTTTGTATGGTGTGCATTGAAGGAGTTTGAAGCTAAAAAAGAGCCAATTAAAGGCTTGTTCTGATAAGTGGT
CCAAATACCTTAAACCTATAGAGCATTCATTACAATAGGAATTAACTTGGTGTTTCAAATTTACTCTGCTGCACAGAAT
AATCCTTCTCCTCTTTAATAATTCAACTCAACTACACCACTACACAGATCTGACATTTCAGAAACTCACAAAAAGAAAA
TGGATGAAAAAGTGGGGAAAAATATTATAGCCTGCAACCTCCATCCATTCTATGGCTGCACTAAAATATCAGAAACTGT
TGGTTTATCTACGGGCACAGTAAAAGTACAGATCCATAGCCTTTGATGAAATGAAACCAACTGCTTCAATTATACATTA
ATCATCATCATCATCGAAAGGCATATTATATGGCTTTAAAGCTTTCTCACACAAACTTGTTTTTGCCTGCCATCGAACC
TACACATGGGGTCACAGTGAAAAAAAAAAAAAAAATAATAATAATAGAATAAAACAAACAAAAACAGGCTTGAATCCATT
CAAGATAGTTTCTGCTATCAACGGAGAAGATTAGGTATTAAATCGAAATCGAATTGAAACCTCACATTTCCGAGTAAAT
TCAGGGAGAAATTGACCTAAAAGTTACCGTTTCTGAAAAGGCAAATTAAGAAACAAACAAACAAACACGCACAAAAACA
GCTTAAGAAAGGGGAAAATTACATCATACGATTTTGTAAGTTACAGAAATATACCGACGGCGCCGGCGATTGAAGGTCA
TTGTAGGCGGCGAATTTCGCTGTGGATTACCGATTTGGTTGACCTTGTCTGTATCTTCCCACAGCTTTATTGAACCGCA
AAAGTTAATGAACAAAGAACGCTTTGAGAGAGAGAGAGAGAGAGAGGAGCGAATTTGGCTCAGGAAGATGCAGTTCAAG
AAAGCTGTGGAAGAATACAAACAGAGCCATTTCTGAAGAACGAAGAAAAGAAAAATTAGGGTATCGAACCCTAGAGAGA
AGGCGGAGACTGGAGGAGAGGAGTGAGA

>L20685
ATTCAACCTCAATGGTGGCCATTTGGGAAAGGGAAGAGGAAGCAACAGGGAAGTGAAGAAGAAATCAAGATTGAGAGAG
AGAGAGAGAGAGAGAAGAAGAAGAAGAAAACCCTAATAATTGTGTGTTTGTATGATGAAAAGAAGTTGTTGGGGTTTGG
GAAGAGCTGACAGAAGAGAGTGAGCACACGCTGCAAGCAATTGTATGAGAAAAGTTCATACCTTTACCAGTTTGTGCTC
ATTTCTCTTTCTGTCAGGCCCCTCTTTCTTCATCTTTTTCTATCATATATCAACACTCCCTCTTCCCCTTTTCTTTCCT
TCATTCTATAACCTTCTTCTTTTTCTTTTTCTAAGGACCCAAACCCGAACAATGAGCATCGAGGAACAAAACAAGGAAG
AAGGGTGTTCGAGCAATGTATATGACGACGACGACGAGCAACGATGCATCACGAGAATGAAAGTAAGTGATGATGT
TGTTTATGAATGAAAGATCTTCTATGTTGCATCATGATATTAAGTCTCATGCATATGTTTGCAATGGTTTATCATTTAT
GTGAGATCTCATATCGGTTGGAGAGAGAAGCGAAACATTCTTTACAAGGGTGTGGAAATCTCTTCCTAGTAGACGTGTT
TTAAAGTTGTGAGGCTAATTGCGATACGTAATGAGCTGAAACGAACAATATCCGATAACGGTGGACTTGGGCAGTTGCA
ATTTCACTTTTTCGTTCTATATAATTTAGGTGAGATCTCACATTAGTTGGAGATGAAAACGAAACATTCTTTATAAAGG
TGTGTAAATTTCTCCCCAGTAGACTCGTTTTAAAGCTGTGAGACTCGAATAACTACTTGAACAATGATGCATGTCTACA
AACTCGGAGAACATAAAGGTGTCATGTGGATAGTCGATGTGTATGAAAGTGATACATACGTGTGCATATGTATTCTAAC
TCAGTAGTGTTTCACTAACTGGATATTTTTAAACTTGTATTTTAGAGTTTATGTTTTACGAT

>L21853
CTAATTTCATCTCAAAAAGGACCATTGCATTAATGAAGAACTAAAGGAACAAAAGAGAGAGAAATTCAGAGTGCGATAG
GCGAAGATGAGAAGAACCAAAGGGTTTGTATTGTATGGCCTGAATTGATGGAAGGTTTTGAGGCAAAAACGAGCCAATT
AAAGGCCCTTCAGTTATGCAGTCAAAATACTCTTAAACATAGCATTGAATACAATGAGAATAATTTCATACTTGACATT
TACTCTGCTACTCAGATTAAGCCTTCTCCACTTTAATAATTCAAGCCACCAACTCTTAAAGAAAAACGATGGAATGAAA
TCTATGGCTGCAGTGAAGACAGATAAAACTGTTAGTTGATCCACGGGCACAGTAAAAGTACAGATCCATAGGCTCTGAA
AGCCGCTTCAATAATAATCATATTCATCATCCAAAACCATAGCTTCAAACAAACCTCACACAAATCTGTTTTTGCCTGC
TATGGAACATACACATGGGGTCACACCAAACTAATAATAATAATACACAAAAAGTAAAATTAAATAAAAACAAAAGCGG
TGTTTGAATCCAGATATCAATGGAGAAGAACAATAGCGATTGAATCAAAATGAACCTAAAATGTGATCTAAAATTACGA
TTTCTGAAAAGTCGGAAGAAAATCGAATGAAAATTGTAAGAAAGAGTTGAGGAACGTTACCGATGGCGGTGGAGGATTG
ATTGCGGAGGCGGAATTTGGTTGGCCCTGTGTGTATCTTCCCACAGCTTTATTGAACCGCAAAAGAAAATGATGAACAA
AAGCGCGCTTTTAAGAGAGAGAGAAGAGCGAATCCGGCTCAACAAGATGCAGTTCAAGAAAGCTGTGGAAGAATACGAA
CAGAGCCATAGGAACAAAATTAGGGCATCAAACCCTAGAACCAGAAAACGGGAGAGGAGCGAGAAAGAGA

>L23344
AAAATTAGAAGTAGCATCTCTCATGGTGTTCCATGGATGGAAGCAATAAACTGAAACCAAATACCTGCAACGATAATAA
ATCTGGATCTATGATACACAAAGTTCTAACAGTTCCGTTATTCCATACGGATTAGAATCGGAGTTTTATGTCCAAAATT
AGATGTAACATCTGTCATTGTGTTTCATGGATGGAAGCAATAAACAGAAACCAAATAACTTCAACGACAATAACTCTAG
CTCTATGATATACAAACATCAGCCTCAATGCGAAACACAGCTAAAGCTCTTCAGTAGTTAACTCCGAAAGCGGACGACA
AAGGAAGAAAGATAATTAAATAAGATTCAGAGCACAGACCAGATCTATTATGTAATTTTCAAACCGGTTATTAATTTCA
GTCATGGAGAGAAATCGAAACTAGCAACAAAGTTCTACAACTCCAATCATGAAAAAATGCGAACAATTAAATTAACTCG
TTCTCTCTCAAAAGTAAGAGAAACTCCGTACCTGAGATCTGTAGGAAGGTAAGAATCCAAGCAATCTTCACCACGGTTT
CCGATTCAGTTGATGCAAGGCGGGATCCAATTCGCCCAAACATTTCCCGAACAAGCTAAACCACACGGCGGCAAAGAAA
TGAAGCAAAAAGTAAAAAAATAGAAGCAGAGAAAAACAGAAAAAGAAGATTAGGAGATGCCGGCCGTCGGCAAACGCAA
ACCGGAGCGAAGCAGTTCCCGACCTGCACCAAGCGAATTAGAGACCGCCGGTGTATAACTGAGAAGAAACAACGCCGAG
ATATTGCCCGCGAAAGATTAAGGATACGGAAATGAGAGGCAAACAGAGAGACTACGGAGATTAAAC

```
>L25150
AAAAAGTGTTGCAGGCAACATTTTTTTGGGTCGATTGCAAGGCTAGATCTTCGGTTTGTGTGTCGGGTGCCCGATCCTC
CTCCATCAACAAAAAAGCTGCAAACTTTGGCTCTCTTTTTGCCCTAATTTTTGTCGTGCGCTTACGACAGTTCAAGCTG
CCAGCATGATCTAACCTTAATCTCTCCTAATTTTCGATTTTCCTGAGGTTCAAGATTAGATCATATGGCAGTTTCACCT
GTTGCTGGCTCCACGAAGAATTCAGCCCTAAATTCAAAGTTCTTGACTATATCAGCCGTTTTCTATCCATAATCCTTAA
AAAAGTGTTGCAGGCAACATTTTTTTGGGTCGATTGCAAGGCTAGATCTTCGGTTTGTGTGTCGGGTGGGACGATAAGG
TAATTGTCTCTTTGATTCGTTCGAGTTATAAAAAGTTTGAATGTAAAACTTTTCTTTTCATTAAAAGGTTTTTGATATC
TCTGTGTATGTATTCATATTTTATAATGCCGATCGAAAGGCTTGAATCGATAAATACTCCGGGTGGTTTATGATTGATG
GGTTTTTCTCATTGCTTATAGTGAGTTATTGTTTTTTTTTCCTAAGCCAATTCCATCCAAGAATGCTATATCTTATCTT
TGTTAGAACCCGATAAGATTAGAGTTTAGAGTTTGAAAATTCGACCTTTTCGAAAATTTTAAGGTTGTTGTGTATTATG
AAAAAATGTAAGGTTGTGTATTATTCGGACTTTAATTTGAAAAATTT

>L25833
TAATCCCATTAAGATGAAGTATTCGAATCACAGTCCCACAGGAGCTTTTAAGATGATGATATATAAGATGGAAAACTTA
TATATACAATACAATACATTTTAGAAAGAAATGATTTCTAAAAAGTTGAAAGCAATAAGCATGGAGAAGGGGAATCTGG
AAAGCACCAAAGCCAAAACTCTTTGGATAAGAGAAATTCTGAGACTCAAAACCCCAACAGTAACCAAAGCTGAAGATCA
ATCTCCATTGCTCTCGCCCCTAACTCTTCCTACGACTAAATTATAAATTAAACCAACCTGCAACACAAGAGAATGAAGA
AGTGTGTTTTTTTGGTATACAGGAATTGGATGAAGCACACAGCAACAAGAAATGAAAATAGGGGGGTTGGGGTTCAATG
ATGGGGAGAAGAAGGGGGATTGGAATATTCGTGATACCACCAAAAGGTGAAGCTACCATATGATCTGATCTTTCCTTGT
GTGAAGAGCAAGAAGAAGTTTAGATCATGCTGGCAGCTTCAACTGCTGGTGATGCACGGCAGAGTAAGAGAGTTTGTGT
TGTAGTAATTTGAAGGAAGAGGAGGGCAATAGGTAAGGGGATATAAATAGAGTTTGTTGCTGTTGATTATTATGTGATT
GCAAGGTTTTGAAGAAAATGAGATGGCAGAGTGGCAGTGGTCCAAAGGTAAGACAAAATTGAATATTATGAAGCCCCAC
GCCATTCACC

>L31431
AATTGAATGATATTTATTTATTTATATTAAATGAAGCTAATCTAAAGGAGTGGTGCATAGGTGGTCGACCTTATCAAAT
ATGGATGCAAAACCCATTGAAAATGACAAGCATGTGACACAACCAAAACACACAAAAAAACCAAACAATATGACGTAAT
AAAACACAATAATTACGTACCGATATAAAATAATAAAGGATAAATCTTCATATCTCCCCACCCGAAGTGATTTTGTGCT
GCTGCTGCTGCTGCTGGCGGCCACTCGCAGCTTCCATGGGCCTTGCAGAAGAAGAAGAGAACCCAAGGTAGCCAAGGAC
GACTTGCCGCCGCGCCCCACATTGACGGCGGCGGCGAGTTGACCCGCCGGCAAGTCATCCTGGGCTGCATTCACCTCCC
TCCTCCTTTGGGAAGGCAGTGGAAGCAACCCAAAAAAACACCATAAACACGTGGTTGGGGTTGGGTTTGGGGTTGGGGT
GTGGGTTTAGGGTGGAGGAT
```

Supplementary Fig. S1. Sequences of transcriptome contigs identified as abundant in the phloem and leaf transcriptomes (Table 1). pre-miRNA regions are shaded, mature miRNA sequences are underlined.
