# Supplementary material for: Identification of miRNA precursors in the phloem of Cucurbita maxima
Source: PeerJ. 2019 Dec 11;7:e8269. doi: 10.7717/peerj.8269 (PMC6911342; doi:10.7717/peerj.8269)
Supplement: Supplemental Information 2 [file peerj-07-8269-s002.doc]

**Supplementary Table S1.**

Primers used for amplification of *C. maxima* pri-miRNAs and TCTP

| **Target sequence** | **Forward primer, 5’ to 3’** | **Reverse primer, 5’ to 3’** |
| --- | --- | --- |
| Detection in *C. maxima* phloem exudate and leaf samples | | |
| pri-miR167b | TTAGTCGTAGGAAGAGTTAGGG | AAGCAATAAGCATGGAGAAGG |
| pri-miR319a | GCTTTCTTCAGTCCACTCAT | GCTCCCTTCAGTCCAAGTA |
| pri-miR396b | TGTGCGTGTTTGTTTGTTTGTTTC | CTGCTATCAACGGAGAAGATTAGGT |
| Detection in grafting experiments | | |
| pri-miR319a | TGAGTCAAAAACCCGACTCTCC | CAAATCTGACTCCAAGCAGACCA |
| TCTP | AGAACATTGAAGGAGCAACTAA | CTCTCAGCACTTGACTTCC |
